# Bacteriophage cocktail for biocontrol of *Escherichia coli* O157:H7: Stability and potential allergenicity study

**DOI:** 10.1371/journal.pone.0195023

**Published:** 2018-05-15

**Authors:** Karina Ramirez, Carmina Cazarez-Montoya, Hector Samuel Lopez-Moreno, Nohelia Castro-del Campo

**Affiliations:** 1 Division de Estudios de Posgrado e Investigacion, Teconlogico Nacional de Mexico-Instituto Tecnologico de Culiacan, Culiacan, Sinaloa, Mexico; 2 Centro de Investigacion en Alimentacion y Desarrollo A.C., Culiacan, Sinaloa, Mexico; 3 CAEC BB-UAS-264, Posgrados de la Facultad de Ciencias Quimico Biologicas, Universidad Autonoma de Sinaloa, Culiacan, Sinaloa, Mexico; Instituto Butantan, BRAZIL

## Abstract

*Escherichia coli* O157:H7 has become a global public health and a food safety problem. Despite the implementation of control strategies that guarantee the safety in various products, outbreaks persist and new alternatives are necessary to reduce this pathogen along the food chain. Recently, our group isolated and characterised lytic bacteriophages against *E*. *coli* O157:H7 with potential to be used as biocontrol agents in food. To this end, phages need certain requirements to allow their manufacture and application. The aim of this study was to determine the physical stability and allergenic potential of free and microencapsulated (ME) bacteriophage cocktails against *E*. *coli* O157:H7. *In vitro* and *in vivo* studies were performed to determine phage survival under different pH, gastrointestinal conditions, temperature and UV light intensities. Results showed that the stability of ME phages was significantly (*P*<0.05) higher than free phages after ultraviolet irradiation, pH conditions between 3 to 7, and exposure to temperatures between at -80°C and 70°C. Both formulations were highly sensitive to very low pH in simulated gastric fluid, but stable in bile salts. *In vivo* studies in mice confirmed these phages passed through the gastrointestinal tract and were excreted in faeces. *In silico*, full-length alignment analysis showed that all phage proteins were negative for allergenic potential, but different predicting criteria classified seven phage proteins with a very low probability to be an allergen. In conclusion, these data demonstrated that microencapsulation provided a greater stability to phage formulation under stress conditions and assure a more suitable commercial formulation for the biological control of *E*. *coli* O157:H7.

## Introduction

Foodborne disease represents a serious public health problem worldwide. The Center for Disease Control and Prevention estimates that each year about 1 in 6 Americans gets sick; 128,000 are hospitalised and 3,000 die from foodborne diseases [1]. Among the major pathogens that cause foodborne illness are *Salmonella*, *Campylobacter*, and *Escherichia coli* O157:H7 [2]. It is estimated that every year 73,000 cases and 61 deaths occur due to *E*. *coli* O157:H7 in the United States [3]; representing a burden cost of 405 million dollars for these infections [4]. Shiga toxin producing *E*. *coli* (STEC) is an emerging pathogen that can cause from diarrhoea to severe diseases like haemolytic uremic syndrome (HUS) [5], a severe complication in which red blood cells are damaged and can cause kidney damage and failure. The high cost of illness caused by STEC (*E*. *coli* O157:H7) infections suggests that additional methods to control this pathogen might be warranted [6]. All these previous facts about the increased number of foodborne outbreaks suggest that methods to inactivate pathogenic bacteria in food are not infallible, which have a negative impact on the health of consumers as well as costs to health systems [7].

Even though there are physical and chemical methods that help minimise the risk, they have not been sufficiently effective, since outbreaks continue to occur. The problem is further aggravated due to antimicrobial resistance of pathogenic bacteria developed as a result of an excessive and inappropriate use of antibiotics and disinfectants. So, it is necessary to have alternatives for the control of pathogenic bacteria such as bacteriophages [8]. These viruses are a biological alternative capable to infect bacteria, replicate and cause cell death and lysis specifically [9,10]. Nowadays, bacterial antibiotic resistance has increased over the last years, being attributed to the overuse and misuse of medications, surging once again interest on phage therapy, and broadening food safety technologies [11,12]. Bacteriophages represent a biological alternative capable of eliminating pathogenic bacteria in food. The advantages of phages offered over antibiotics are the narrow host range and less serious side effects as compared to fatal allergies induced by antibiotics.

In recent years, phages with lytic potential have been isolated and used as agents for decontamination of food products. Phage used for these purposes must meet certain requirements to be applied in food among which include being exclusively lytic, have a broad host range (infecting the desired species or genera), storage stability and application, selection to exclude prophage carriage and risk of transduction, and no known genomic sequence associated with pathogenicity or potentially allergenic proteins [13,14].

The effectiveness of bacteriophages application as biological control of pathogens depends on several factors and environmental conditions [15]. Adverse factors such as temperature, salinity, pH, UV light and gastrointestinal conditions, affect the viability and storage of bacteriophages under storage [16]. Therefore, determining the sensitivity of bacteriophage to external factors is useful and represents a challenge for the development of phage based products that can be used in applications for pharmaceutical and food industries [17,18].

In addition, within the requirements to be met by phage-based products to be used in food for human consumption, they should not induce adverse effects on human health and be generally recognised as safe (GRAS). Therefore, studies should be conducted to validate their safety for the consumer.

Bacteriophages have demonstrated efficacy in reducing bacterial populations and therefore represent a potential alternative for the control of pathogenic bacteria. For this reason, we evaluated the stability of two anti-*E*. *coli* O157:H7 bacteriophage formulations, free and microencapsulated phages, under unfavourable conditions, during storage and application, and the allergenicity potential to broaden the application forms that allow bacteriophages be a viable option for reducing foodborne illness.

## Materials and methods

### Bacterial strains and bacteriophages

Bacteriophages ΦJLA23, ΦKP26, ΦC119 and ΦE142 were provided by the National Laboratory for Research in Food Safety (LANIIA CIAD, Sinaloa, Mexico)[19,20]. *E*. *coli* EC-48 (63-Fv18-1) was used for phage propagation and plaque count assays. Twenty-three *E*. *coli* O157:H7 strains [21] and two reference strains, *E*. *coli* O6 ATCC 25922 and *E*. *coli* ATCC 15597, were used for the host range determination. All bacterial cultures were grown in EC medium (Difco-BD, Mexico) at 37°C for 18–24 h.

### Phage propagation

Each phage was propagated on *E*. *coli* (63-Fv18-1) as described previously by Carey-Smith and colleagues [22] with modifications. Briefly, overnight *E*. *coli* cultures (1 mL) were added to 3 mL of Trypticase soy broth with 4% agarose (TSB-agarose) (Difco-BD, Franklin Lakes, NJ) at 45°C and mixed with 100 μL of phage. The mix was poured onto Trypticase soy agar (TSA) plates (Bioxon, Mexico) and incubated for 18 to 24 h at 37°C. The phages were centrifuged at 4,000 × *g* for 15 min at 4°C and the supernatants were sterile filtered through a 0.45 μm sterile membrane filter (Whatman, USA). Phage stocks were further concentrated by centrifugation (24,446 × *g*, 3 h, 4°C), resuspended in 10 mL of SM buffer [8 mM MgSO_4_, 100 mM NaCl, 0.01% (w/v) gelatine and 50 mM Tris-HCl (pH 7.5)] and maintained at 4°C. All phage titres for all assays were determined by the double-layer plaque assay as described previously [23].

### Free phages cocktail

The free phages cocktail was prepared with the phages ΦJLA23, ΦKP26, ΦC119 and ΦE142 at a concentration of 10^9^ PFU/mL per phage. The phage cocktail titre was determined.

### Microencapsulated phages

The microencapsulated phages contained a polymer mixture consisting of 10% solids and 60% of SM buffer and 30% of bacteriophages cocktail (ΦJLA23, ΦKP26, ΦC119, ΦE142). To microencapsulate phages, first the polymers (modified starch [Capsul^®^; 10% moisture, pH 3 and 90% solubility] and maltodextrin [Sigma-Aldrich^®^; 6% humidity, pH 4–5.5]) were mixed in SM buffer with constant stirring. Then, the polymer solution was stored at 4°C for 15 min and the bacteriophages cocktail (10^9^ PFU/mL) was added stirring gently. The polymer-bacteriophages mixture was spray-dried in a Mini Spray Dryer Büchi, model B-191 (Büchi, Labortechnik AG, Flawil, Switzerland). After drying the entire mixture, the microencapsulated phages (1 g) were placed in a sterile bottle containing 9 mL of SM buffer to subsequently determine phage titre. The phage microencapsulation process had an efficiency of 99% and the average sizes of microcapsules were ~3 μm. The microencapsulated phages were maintained at 4°C [24].

### Host range determination and *in vitro* challenge

The host range of the phage cocktail was determined using 23 strains of *E*. *coli* O157:H7 [21] and two *E*. *coli* reference strains (ATCC 25922 and 15597), while for the *in vitro* challenge an *E*. *coli* O157:H7 was used [21]. Both assays were performed according to the method described by Jamalludeen and colleagues [25] with some modifications. Briefly, bacterial strains were cultured in 5 mL of TSB at 37°C overnight with constant agitation (100 rpm/min) and harvested by centrifugation at 13,800 ×*g* for 10 min. The bacterial pellet was resuspended in phosphate-buffered solution, and 1 mL of each bacterial strain was individually poured into a tube containing 3 mL of 0.4% top agarose at 48°C. The suspension was transferred to a Petri dish with a TSA layer and solidified [22]. Then, 10 μL of each phage suspension (10^10^–10^12^ PFU/mL) was spotted on the overlay surface, dried, and incubated for 22 ± 2 h at 37°C. After incubation, bacterial reduction was calculated as a percentage for the *in vitro* challenge, while the host range was determined based on the bacterial susceptibility profile to the phage cocktail.

### Biocontrol on tomato surface

For this purpose, the immersion method was performed as previously described by Chaidez and colleagues [26] with some modifications. Briefly, 72 tomatoes were divided into two groups: the first group consisted of 36 *E*. *coli* O157:H7-inoculated tomatoes. The second group had 36 microencapsulated phage cocktail-inoculated tomatoes and then sprayed with *E*. *coli* O157:H7; the tomatoes were placed in a rotary mixer spin (Rotamix RKVSD, ATR, MD, USA) at a speed of 60 rpm and sprayed from a distance of 40 cm for 45 s at 25°C with 2 mL of the bacteria [27]. A third group (control) was not inoculated with anything. Then, all tomatoes were dried for 1 h on parallel sterile glass rods placed in a biosafety cabinet and stored in sterilised bags (Johnson USA) at 4°C until use [28]. Following bacteria and bacteriophage inoculation, *E*. *coli* O157:H7 concentrations were analysed on tomatoes every 24 h for 5 days. Two independent experiments were performed with 3 replicates per experiment.

### Temperature stability assay

To determine the effect of temperature on phages, the method described by Basdew and Laing [29] was performed with modifications. Briefly, samples of 1 mL or 1 g of phage preparation (10^9^ PFU/mL or PFU/g) were placed into a water-bath at 50°C and 70°C. Aliquots of 100 μL at different timepoints (0, 30, 60 and 90 min) were immediately assayed for phages survival. Phages incubated at 25°C were used as a control. To determine the sensitivity of phages to low temperatures, 1 mL of free phages (6 × 10^9^ PFU/mL) or 1 g of microencapsulated phages (4 × 10^9^ PFU/g) were maintained at temperatures of -20°C and -80°C at different timepoints (0, 1, 7, and 30 days). For long-term storage, 1 g of microencapsulated phages were maintained at 4°C for 4 years. Bacteriophage survival was calculated with the equation: % Survival = (Nt/No) × 100, where No indicates the initial concentration of bacteriophages before treatment and Nt, the final concentration of bacteriophages after treatment. All assays were carried out in triplicate.

### UV radiation stability assay

Microencapsulated and free phages were exposed to UV radiation using the methodology described by [30] with modifications. Briefly, samples of free (2 mL) or microencapsulated (1 g) phages were placed in sterile petri dishes and irradiated for 0, 15 and 30 min with a UV lamp (λ = 254 nm) in a laminar flow cabinet at a distance of 0.6 m. Aliquots were removed at each sampling timepoint and phage survival was calculated.

### pH stability assay

pH stability was determined using the methodology described by Ma and colleagues [31]. Briefly, samples (100 μL or 100 mg) of free (10^8^ PFU/mL) or microencapsulated phages (10^9^ PFU/g) were added to 9.9 mL of prewarmed at 37°C in 0.2% (w/v) NaCl and adjusted to different pH levels (2, 3, 6, 9 or 10). After 5 min incubation, free and microencapsulated phages titres were calculated as described above. The assay was replicated three times, and the results are reported as phage survival percentage.

### Stability in simulated gastric fluid (SGF)

Free and microencapsulated phages (10^9^ PFU/g) were exposed to SGF containing 3.2 mg/mL pepsin in 0.2% (w/v) NaCl at pH 2.0 and 2.4. Microencapsulated (100 mg) or free (100 μL) phages were added to test tubes containing 9.9 mL of prewarmed (37°C) SGF and incubated for 0, 5, 15 and 30 min at 37°C. SM buffer (pH 7.5) was used as a control. After each timepoint, the titre was calculated. The experiment was replicated three times, and results are shown as phage survival percentage [31].

### Stability in bile salts (BS)

Samples of 100 mg of microencapsulated (4 × 10^9^ PFU/g) or 100 μL of free phages (6 × 10^9^ PFU/mL) were added to 9.9 mL of prewarmed (37°C) simulated bile [1 and 2% (w/v) porcine bile extract] (Sigma-Aldrich, Oakville, ON, Canada), and incubated for 1 h and 3 h at 37°C. After incubation, 100 μL-aliquots were collected, and phage titres were obtained as described above. Distilled sterile water at pH 7 was used as control. The assay was replicated three times, and results were reported as phage survival percentage.

### Phage inactivation kinetics in simulated intestinal fluid (SIF)

Samples (200 mg) of microencapsulated phages were added to conical tubes with 50 mL of prewarmed SIF [32] and incubated at 37°C with shaking at 100 rpm. Titres were determined 0, 2, 5, 15, 30 and 60 min after incubation. Controls included microencapsulated phages incubated in Phosphate-Buffered Saline (PBS). After each time point, 100 μL-aliquots were collected and phage titres were determined by the double-layer plaque assay. The assay was replicated three times, and the results are reported as the phage release percentage calculated according to the equation RP (%) = (C_F_/C_T_) × 100, where RP is phage release, C_T_ is the total microencapsulated cocktail concentration and C_F_ is the concentration of free phages.

### *In vivo* phage survival

BALB/c mice (8 to 10-week-old) were orally administered (gavage) with a single dose (100 μL) of different concentrations of free or microencapsulated phages in mineral water. Mice were randomly assigned in four groups (n = 5) depending on phage concentration: I (10^3^ PFU/mL); II (10^6^ PFU/mL); III (10^10^ PFU/mL) and IV (mineral water). After seven days, faecal samples (0.1 g) were collected in eppendorf tubes and suspended in 0.9 mL of SM buffer, homogenised by shaking and centrifuged (9,500 × *g*, 10 min, 4°C). Phage titres were quantified in the supernatant by double-layer agar method [33]. For *E*. *coli* quantification, 0.1 g of faecal material was serially diluted in peptone water (0.1% w/v) and bacteria counts were enumerated by serial dilutions in Chromagar ECC (Paris, France). Prior to phage administration, basal levels of *E*. *coli* and lytic phages were quantified in each mouse. All efforts were made to minimize animal discomfort and suffering. Mice were sacrificed by cervical dislocation. The experimental protocol was approved by the Center for Research in Food and Development (CIAD, Centro de Investigacion en Alimentacion y Desarrollo) Animal Ethics Committee (CE/016/2015) and complied with the Official Mexican Normative Guidelines NOM-062-ZOO-1999 Technical specifications for the production, care and use of laboratory animals and the guide for care and use of laboratory animals [34].

### *In silico* evaluation of allergenicity

Predicted gene products of phage ΦJLA23 (Genebank KC333879.1), ΦKP26 (Genebank KC579452.1), ΦE142 (Genebank KU255730.1) and ΦC119 (Genebank KT825490.1) were screened by an *in silico* analysis according to FAO/WHO guidelines for possible similarities to currently known protein food allergens using online tools at the Food Allergy Research and Resource Program (FARRP) allergen database [35]. Additional analyses were performed to proteins with hits in the FARRP-sliding 80mer FASTA (FAO/WHO >35%) criterion using the following tools: AllergenFP [36], AllerTOP [37], Proinflam [38], SDAP [39], AlgPred [40] and PREAL^w^ [41].

### Statistical analysis

All parameters were compared using a two-way analysis of variance (ANOVA) followed by Tukey´s test for multiple comparisons among groups. Each experiment was replicated three times. Differences with *P*<0.05 were considered significant at 95% confidence interval. Statistical analysis was performed using Minitab 16.0 (State College, PA).

## Results

### Phage cocktail efficacy

**Fig 1:**
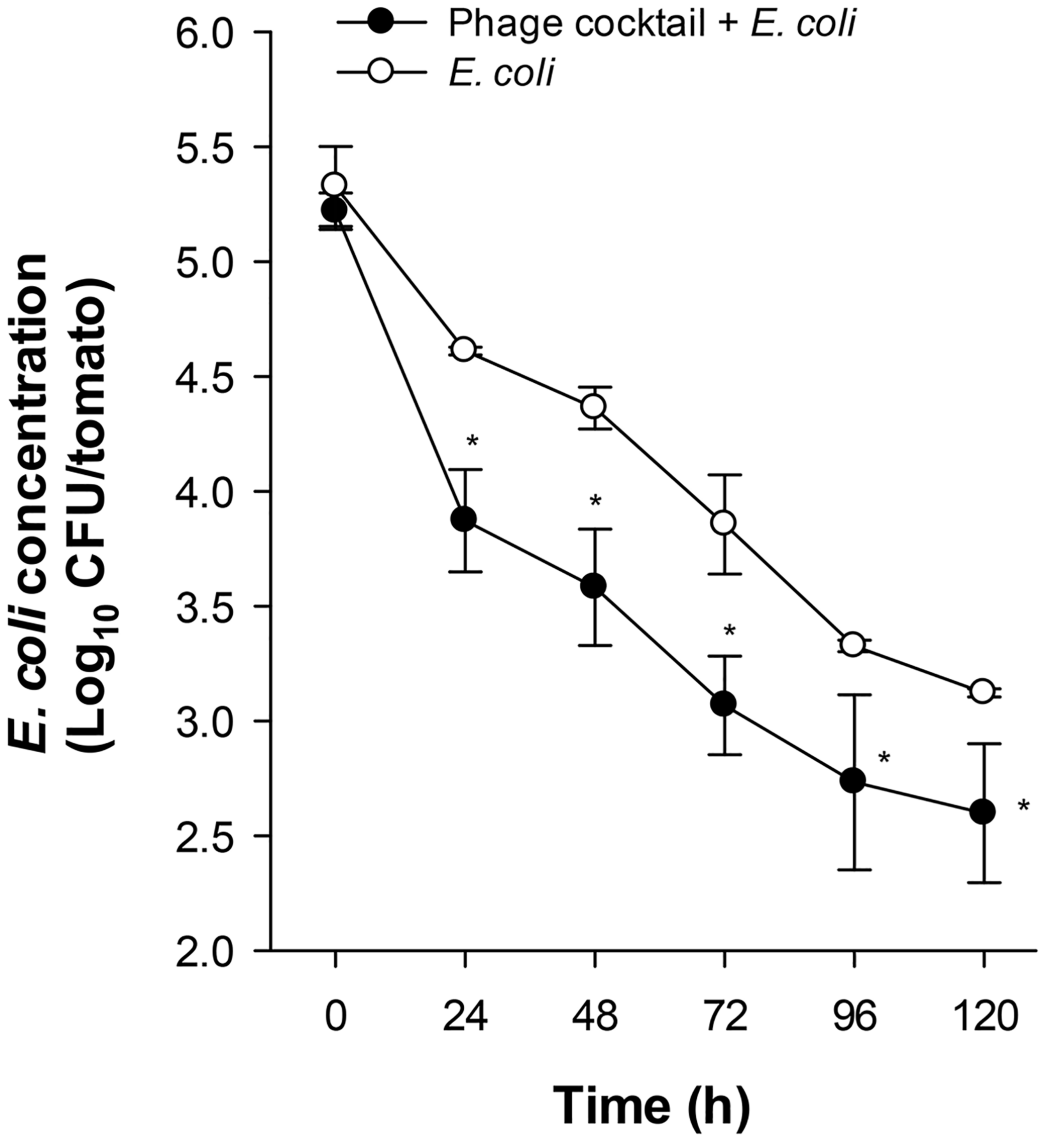
Phage cocktail biocontrol on tomato surface. Tomatoes were sprayed with the microencapsulated phage cocktail and then inoculated with *E*. *coli* O157:H7 and stored at 4°C for different times. Data represents mean values ± SEM of two independent experiments. *, *P*<0.05 compared to tomatoes non-treated with the microencapsulated phage cocktail.

Here, we examined two candidates anti-*E*. *coli* O157:H7 bacteriophage formulations to be used for biocontrol in food, one prepared as a cocktail of free phages and other as a cocktail of microencapsulated phages, henceforth referred as free and microencapsulated phages. First, we assessed the lytic effect of a phage cocktail against *E*. *coli* O157:H7 by a bacterium/bacteriophage *in vitro* challenge in liquid culture. *E*. *coli* O157:H7 was reduced 99 ± 4% (~ 2 log_10_) after 60 min of the phage cocktail infection. Then, the phage cocktail host range was tested for lytic activity on twenty-three *E*. *coli* O157:H7 isolates [21] and two *E*. *coli* reference strains. Twenty-one strains of the 23 *E*. *coli* O157:H7 isolates were susceptible to the phage cocktail, while the two reference strains were not lysed. An *in vivo* challenge on the tomato surface was also performed to demonstrate the biocontrol efficacy of the microencapsulated phages on *E*. *coli* O157:H7. The microencapsulated phages were sprayed onto the tomato surface and then inoculated with the bacteria and stored at 4°C during 5 days (Fig 1). After 24 h, phage-treated tomatoes significantly decreased *E*. *coli* O157:H7 concentration as compared to the control group of tomatoes that did not receive the phage cocktail. These differences in *E*. *coli* O157:H7 reductions were maintained until day 5. These results indicate that the phage cocktail is host specific and able to lyse *E*. *coli* O157:H7 *in vitro* and on tomato surface.

### Temperature stability

**Fig 2:**
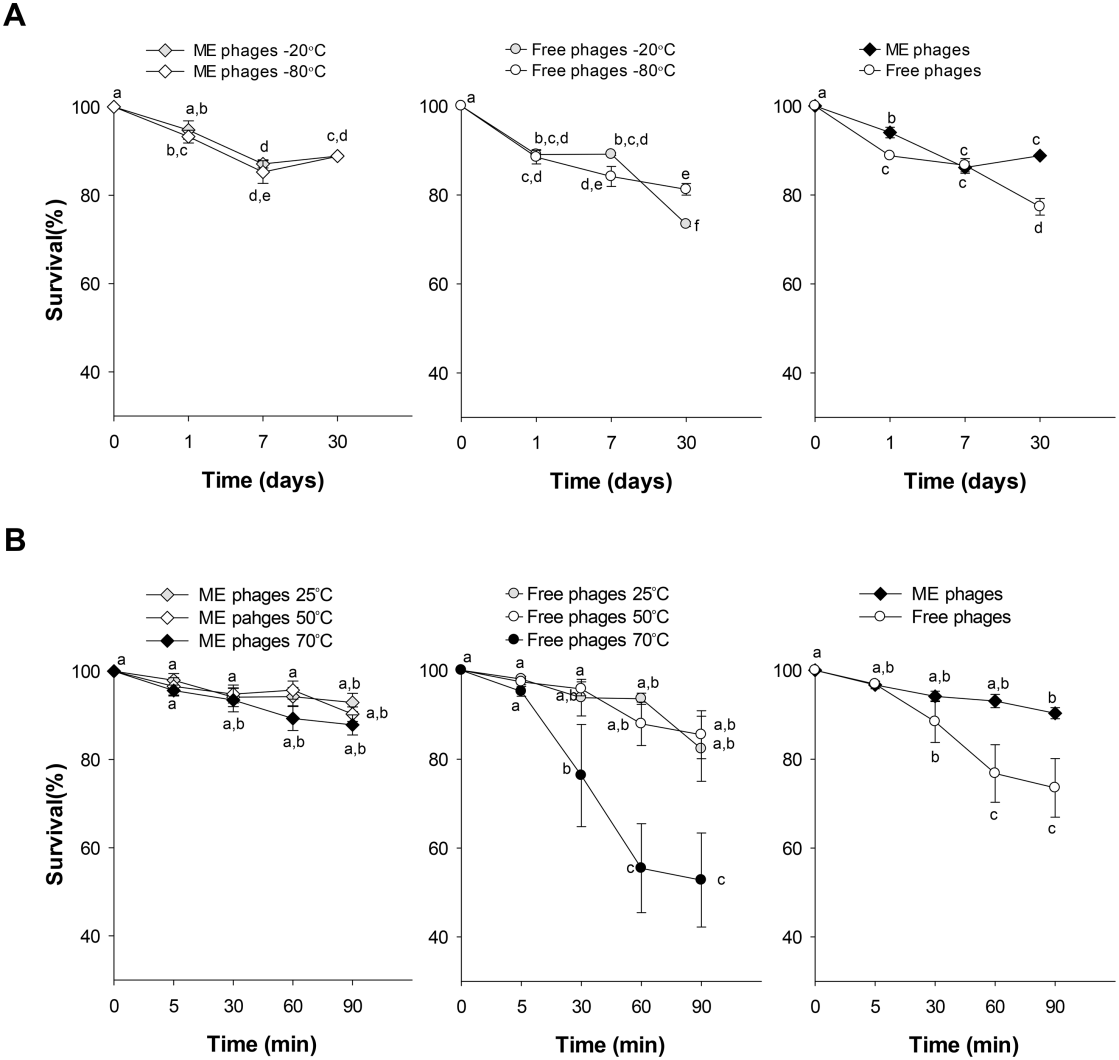
Stability of microencapsulated and free phages at different storage temperatures. Microencapsulated (ME) and free phages were stored at low (A) and high (B) temperatures for different times. Data represents mean values ± SEM of three independent experiments. Right, interaction plot of phage survival means calculated for timepoint at each phage condition. Different letters represent significant differences between groups (*P*<0.02), whereas letters shared in common between or among the groups indicate no significant differences.

To use bacteriophages as biocontrol products in food, it is important to evaluate the product physical stability for potential manufacture. One preservation method for long-term storage includes freezing. Free and microencapsulated phages were maintained at freezing temperatures (-20°C and -80°C) for different time intervals (0, 1, 7 and 30 days) and the phage survival rate was determined (Fig 2A). For both temperatures, microencapsulated phages maintained a survival rate within 89% (~0.05 log_10_ reduction) after 30 days of storage, while free phages significantly decreased over time (*P*<0.001). Even when the lytic activity of free phages was reduced, they exhibited survival rates of 73% (~0.14 log_10_ reduction) and 81% (~0.09 log_10_ reduction) at -20°C and -80°C. The interaction analysis between formulation, time and temperature after storage at -20°C and -80°C showed significant differences (*P* = 0.010), while no differences were observed between temperatures. In addition, when microencapsulated phages were maintained at 4°C for 4 years, the phage survival rate was maintained within ~50% (~0.32 log_10_ reduction).

To analyse the stability of phages at high temperatures free and microencapsulated phages were exposed to 50°C and 70°C (Fig 2B). The survival rate of microencapsulated phages was highly stable with 88% (~0.05 log_10_ reduction) after being exposed for 90 min at 70°C. On the contrary, when the exposure time increased for free phages, the lytic activity started to decrease after 30 min and after 90 min at 70°C was reduced 53% (~0.32 log_10_ reduction). Significant differences were observed in the interaction between formulating factors, temperature and time (*P*<0.001). No differences were found between the mean survival at 25°C and 50°C for both formulations. Survival of microencapsulated phages is not adversely affected by exposure to high or low temperatures, whereas free phages denote greater sensitivity to high temperatures such as 70°C.

### Effects of UV irradiation on phage cocktails

**Fig 3:**
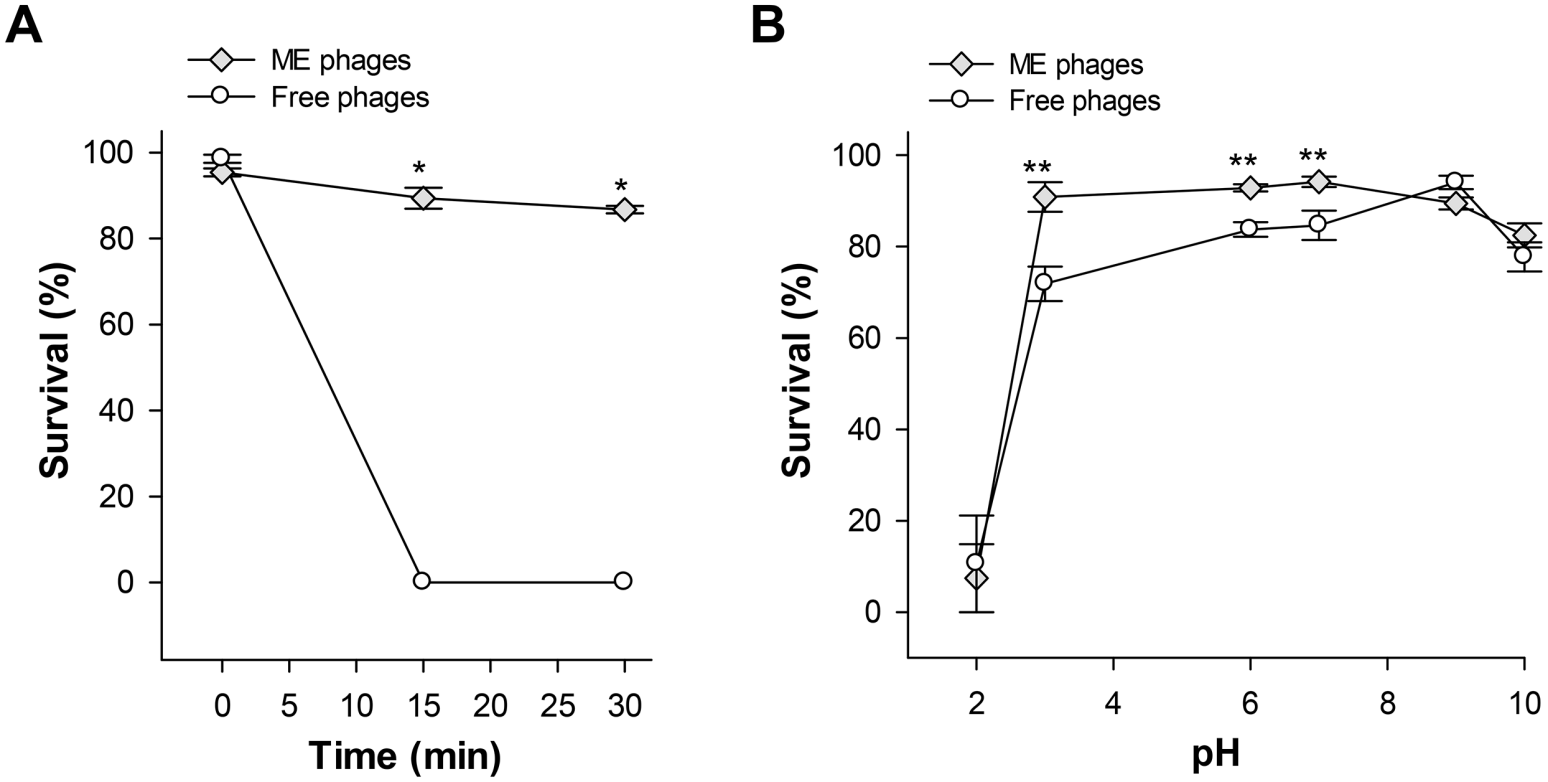
Effects of UV light and pH on the survival of microencapsulated and free phages. Microencapsulated (ME) and free phages were exposed to UV light for 15 and 30 min (A) or incubated at different pH values for 5 min at 37°C (B). Values are expressed as the phage survival percentage and represents mean ± SEM of three independent experiments. *, *P*<0.001; **, *P*<0.05 compared to free phages.

Free and microencapsulated phages were exposed to UV light irradiation for 15 and 30 min. We observed that microencapsulated phages were slightly affected after 15 and 30 min (90% phage survival rate, ~0.04 log_10_ reduction), while free phages showed undetectable levels after 15 min of exposure (*P*<0.001) (Fig 3A). These results suggest that microencapsulation provides protection against the effect of UV light and improves stability.

### pH stability

To survive, bacteriophages must be resistant to acid or alkaline environments. Free and microencapsulated phages were subjected to various pH conditions (2, 3, 6, 7, 9 and 10) for 5 min at 37°C (Fig 3B). Results showed significant differences in the survival rate of free and microencapsulated phages to different pH values (*P*<0.001). Microencapsulated phages retained significant higher lytic capability than free phages at pH ranging from 3 to 7 (*P*<0.05); however, at pH 2, free and microencapsulated phage concentrations decreased by 89% (~0.96 log_10_ reduction) and 93% (~1.15 log_10_ reduction). No differences were observed between the two cocktails at pH 9 and 10. These results suggest microencapsulation may confer acid resistant properties to phages.

### Bacteriophages tolerance to simulated gastric fluid

**Fig 4:**
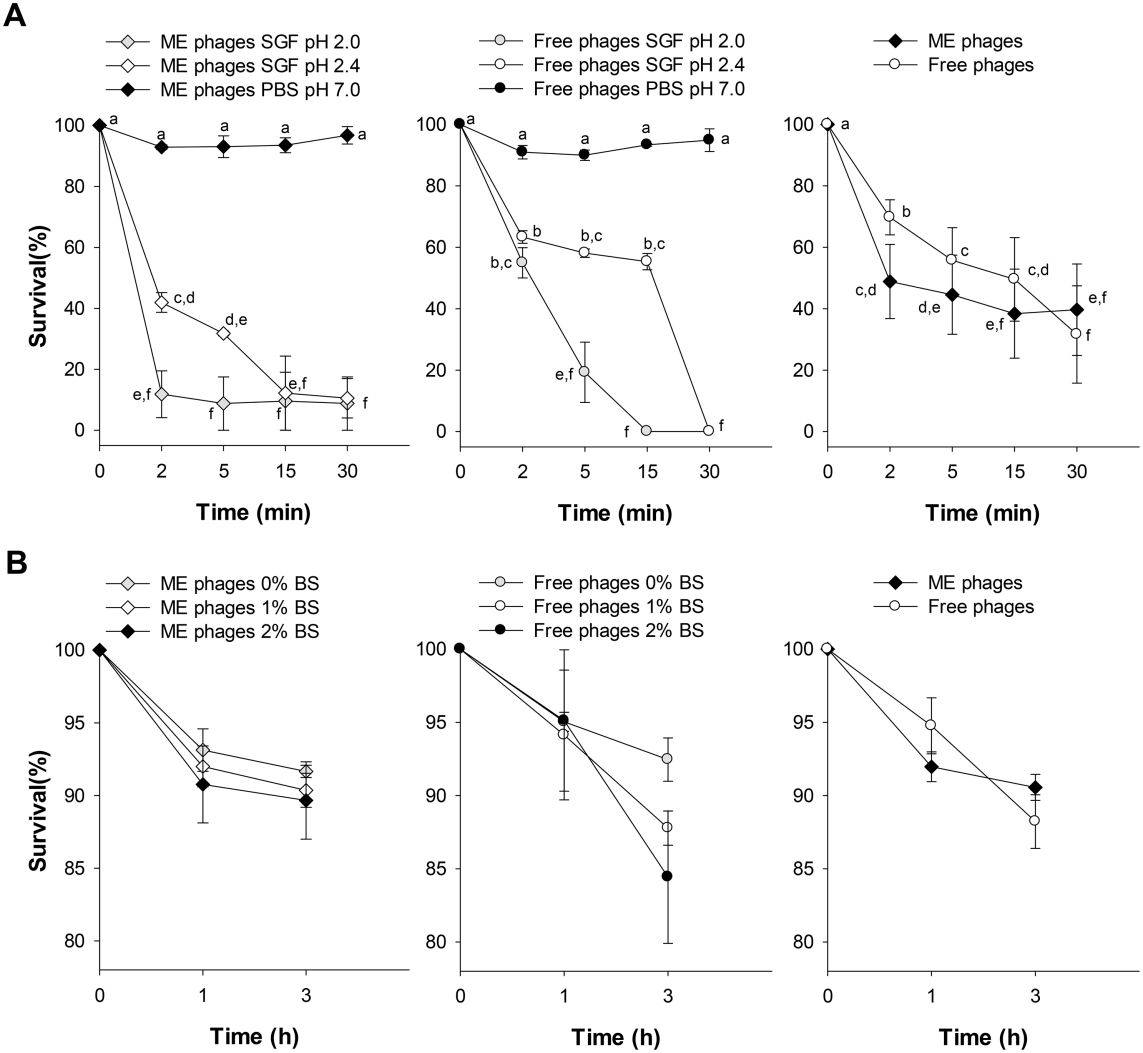
Effects of simulated gastric fluid (SGF) and bile salts (BS) on the survival of microencapsulated and free phages. Microencapsulated (ME) and free phages were exposed to SGF (A) at different pH values for 2, 5, 15 and 30 min or exposed to BS (B) at 1% and 2% for 1 and 3 h. Data represents mean values ± SEM of three independent experiments. Right, interaction plot of phage survival means calculated for timepoint at each phage condition. Different letters represent significant differences between groups (*P*<0.05), whereas letters shared in common between or among the groups indicate no significant differences.

To determine the survival of bacteriophages passage through the stomach, both formulations were subjected to simulated gastric fluid (SGF) at pH 2 and 2.4. Microencapsulated and free phages showed the lowest viability to SGF at pH 2. Interestingly, contrary to expectations, free phages exposed to SGF at pH 2.4 showed higher viability after 2, 5 and 15 min compared to microencapsulated phages (Fig 4A). However, after 30 min, free phages were reduced to undetectable levels, while 9% (~1.04 log_10_ reduction) of microencapsulated phages were still detected. Statistical analysis showed significant differences between the interaction of the formulation type, pH values and time intervals (*P*<0.001).

### Bacteriophages stability in bile salts

Both bacteriophage formulations had high resistance to 1 and 2% bile salt concentrations after 3 h of incubation at 37°C. Results showed that free and microencapsulated phage concentrations were reduced by 0.8 log_10_ and 0.7 log_10_ after 3 h of incubation at 37°C (Fig 4B). Statistical analysis showed no significant difference between bile salt concentrations and the formulation type, but there was a significant difference in time (*P* = 0.028).

### *In vitro* release of the microencapsulated phages in simulated intestinal fluid (SIF)

Microencapsulated phages (4 × 10^9^ PFU/g) were added to 50 mL of prewarmed SIF at pH 6.8 and kinetics of phage release were determined (Table 1). After 2 min of incubation in SIF, phage release was nearly 50% and remained similar to 67 ±0.5% after 5, 15, 30 and 60 min. Apparently, microencapsulated phages incubated in SIF were released significantly slower than phages in PBS (*P*<0.001), but it is uncertain whether phages are not being released or it is actually due to the enzymes inactivating them.

**Table 1:** ME phage cocktail stability in simulated intestinal fluid.

Time (min)	Bacteriophage release (%)	
	PBS	SIF
0	0.0 ± 0.00	0.0 ± 0.00
2	92.9 ± 1.33	53.5 ± 3.08^a^
5	93.1 ± 6.17	66.7 ± 1.03^a^
15	93.5 ± 4.22	67.4 ± 0.98^a^
30	96.8 ± 4.94	66.1 ± 1.05^a^
60	94.9 ± 6.39	66.8 ± 0.65^a^

PBS, Phosphate buffered-saline; SIF, Simulated intestinal fluid.

^a^*P*<0.001 compared to the PBS group.

### *In vivo* release of the microencapsulated bacteriophages

**Fig 5:**
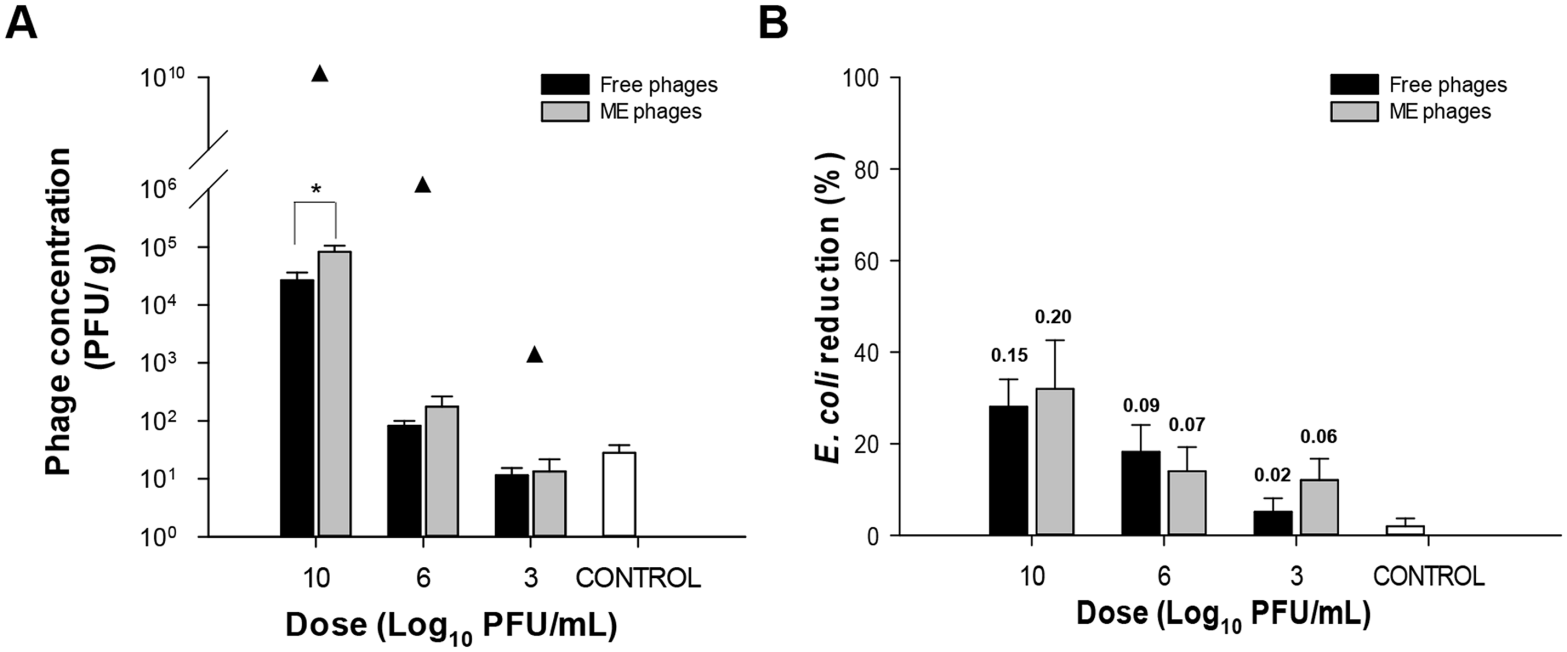
Phage survival after oral administration. Mice (n = 5 per group) were orally exposed to a single dose of 10, 6, 3 log_10_ PFU/mL of the free or microencapsulated phage cocktails. The control group included mice treated with mineral water. (A) Faecal shedding after seven days of the administration of the free or microencapsulated phage cocktails at different doses. Results are expressed as mean phage titres ± SEM of two independent experiments. *, *P*<0.02 compared to free phages. Closed triangles indicate mean phage dose plus baseline phage titres. (B) Percentage reduction in *E*. *coli* shedding in free or microencapsulated phage cocktail-treated mice on day 7 was determined relative to baseline counts found in mice before treatment. Values represent mean concentration ± SEM of two independent experiments. Numbers above bars indicate mean log_10_ CFU/g of *E*. *coli* reduction. No significant differences were observed between groups.

To determine the shedding of free and microencapsulated bacteriophages after oral administration, four groups of BALB/c mice (n = 5) were treated with a single dose of different phage concentrations and phage survival was measured in mice faeces (Fig 5). Mice treated with the highest concentration of microencapsulated phages exhibited higher phage counts compared to mice treated with the free phage cocktail (*P*<0.02, Fig 5A). However, groups administered with lower doses showed no significant differences between both phage cocktails. These results suggest that the free and microencapsulated phage cocktails were able to survive the passage through the gastrointestinal tract.

Intestinal *E*. *coli* is part of the normal microbiota of certain mammals and could contribute to phage replication; therefore, bacterial concentration in the intestine could be affected. For this reason, *E*. *coli* concentrations in mouse faeces were quantified before and after the administration of different doses of the free and microencapsulated phage cocktails to determine bacterial reductions (Fig 5B). In general, baseline *E*. *coli* concentrations in faeces were low (2.9 ± 2.0 log_10_ CFU/g). After phage cocktail administration, bacterial concentration in faeces of mice that received the higher dose of free and microencapsulated phage cocktails exhibited the highest log_10_ CFU/g reduction of counts in mice faeces (0.15 log_10_ CFU/g and 0.20 log_10_ CFU/g, respectively); however, no significant differences were observed in bacterial reductions between all treated groups or the control.

### Determination of potential allergenicity of bacteriophage proteins

To determine possible potentially allergenic proteins, the amino acid sequences of all proteins of the four phages were compared *in silico* to food allergenic protein sequences registered in the FARRP database version 13. Analyses were performed based on the full-length FASTA criterion and the sliding 80mer FASTA-FAO/WHO criterion that defines a probable allergen as a protein that exhibits a local similarity match of 35% over 80 amino acids [35]. All proteins of the four phage did not show hits for the full-length FASTA criterion based on an identity greater than 50% and an E score less than 1e-7 (Table 2)[19,20].

**Table 2:** *In silico* analyses of phage proteins potential allergenicity.

Phage	ΦJLA23	ΦE142	ΦKP26	ΦC119
**Total Proteins**	65	193	78	74
**AllergenOnline**				
• Full-length FASTA (Identity >50% and E score < 1e-7)	No hits	No hits^1^	No hits	No hits^2^
• Sliding 80mer FASTA (FAO/WHO >35%)	One protein	One protein	Two proteins	Three proteins

FAO, Food and Agriculture Organization of the United Nations; WHO, World Health Organization.

^1^[19].

^2^[20].

For the sliding 80mer FASTA analysis, phages ΦJLA23 and ΦE142 showed one protein with hits, while phage ΦKP26 and phage ΦC119 had two and three proteins, respectively. Phage protein sequences with hits were further analysed with additional tools (Table 3). The AlgPred prediction tool, based on the integration of various approaches to predict allergenic proteins with high accuracy, and the motif-based prediction by PREAL^w^ classified these same proteins as probable non-allergens.

**Table 3:** *In silico* analysis of phage proteins with sliding 80mer FASTA hits (FAO/WHO >35%).

	ΦJLA23	ΦE142	ΦKP26		ΦC119		
Phage protein	Putative tail length tape measure protein precursor	Tail Fiber adhesin	Putative DNA polymerase I	Tail tape measure protein	Putative DNA polymerase I	Putative tail length tape measure protein precursor	Hypothetical protein
**AllergenFP**	-	-	-	+	-	+	+
**AllerTOP**	-	-	-	-	-	-	+
**Proinflam**	No hits	No hits	No hits	No hits	No hits	No hits	No hits
**SDAP Full-length FASTA (E score < 1e-7)**	0	0	3	0	3	0	0
**AlgPred**	-	-	-	-	-	-	-
• MAST result	-	-	-	-	-	-	-
• Prediction by mapping of IgE epitope	Does not contain	Does not contain	Does not contain	Does not contain	Does not contain	Does not contain	Does not contain
• BLAST result of ARPs	-	-	-	-	-	-	-
• Prediction by hybrid approach (SVMc +IgE epitope +ARPs +BLAST +MAST)	-/+	-/-	-/+	-/+	-/+	-/+	-/+
**PREAL**^**W**^	-	-	-	-	-	-	-
• FAO/WHO sequence alignment	+	-	-	+	-	+	-
• FAO/WHO aminoacids match	-	-	-	-	-	-	-
• Motif based	-	-	-	-	-	-	-

-, Probable non allergen;

+, probable allergen;

-/+, non allergen-allergen;

-/- non allergen-non allergen;

Allergen FP, Bioinformatics tool for allergenicity prediction based on a fingerprint approach; AllerTOP, Bioinformatics tool for allergenicity prediction; Proinflam, Prediction of proinflammatory epitopes; SDAP, Structural Database of Allergenic Proteins; AlgPred, Prediction of Allergenic Proteins and Mapping of IgE epitopes; MAST, Motif Alignment and Search Tool; BLAST, Basic Local Alignment Search Tool; IgE, Immunoglobulin E; ARPs, Allergen Representative Peptides; SVMc, Support Vector Machine modules; PREAL^W^, Allergen prediction based on weighted average score; FAO, Food and Agriculture Organization of the United Nations; WHO, World Health Organization.

However, the Allergen FP prediction method based on a fingerprint approach classified the tail tape measure protein of phage ΦKP26, the putative tail length tape measure protein precursor and the hypothetical protein of phage ΦC119 as probable allergens. This last protein was also classified as a probable allergen by the Allertop prediction tool, based on the comparison of protein sequences with known and non-allergens. Furthermore, putative DNA polymerase I of phages ΦKP26 and ΦC119 showed three hits in the full-length FASTA-SDAP analysis. AlgPred prediction, based on a hybrid approach that allows to predict probable allergens using combined parameters (SVMC, IgE Epitope, ARPs, BLAST, MAST) classified the putative tail length tape measure protein precursor of phages ΦJLA23 and ΦC119, the putative DNA polymerase I of phages ΦKP26 and ΦC119, and the hypothetical protein of phage ΦC119 as probable allergens and non-allergens. Tail fibre adhesion protein of phage ΦE142 was classified as non-allergen under the same criteria.

Finally, all four phages proteins exhibited no hits for antigenic regions that induce an inflammatory response and did not contain IgE epitopes.

## Discussion

Products that can improve food safety within the food chain are demanded. Bacteriophages represent a biological alternative that could be applied from primary production, during processing, storage and display; and impacts in agricultural, industrial and commercial sectors. However, bacteriophage applications and their versatility are determined by their sensitivity to physical factors due to their limited ability to survive to diverse hostile environmental factors. To this end, bacteriophage candidates intended for use in the food chain need to be evaluated in several parameters to assess their application feasibility. In this study, we evaluated several properties of two phage cocktails against *E*. *coli* O157:H7 (free and microencapsulated phages) as candidate products to be applied in the food chain.

Temperature tolerance represents a product stability advantage in shelf life, and bacteriophages require being tolerant to temperature variations that may occur by climate or storage conditions. In our study, microencapsulated phages were more stable than free phages when stored at -80°C for a month with minor viability reductions. A recent study reported the stability of microencapsulated phages against *Salmonella* after three and six months storage with low reductions in phage viability [42]. Phage viability decrease induced by low temperatures to free phages (25%) for long periods has been observed for other phages, such as MS2, which was inactivated by 75% after a storage up to 300 days at -80°C [43]. Another study, showed phage P100 reduction by six to seven log_10_ at 4°C after 2 weeks exposure [44]. Furthermore, in our study, exposure to high temperatures did not adversely affect microencapsulated phage survival, whereas free phages denoted greater sensitivity at 70°C. These results are consistent with previous studies that demonstrated the reduction in lytic phage ability after prolonged exposure to high temperatures [29,45]. Liu and colleagues [46] observed that bacteriophage type S13 infectivity was decreased at 45°C and 55°C, but survival was not detected after 30 min exposure at 70°C. This reduction is mainly attributed to phage protein denaturation and damage to the physical structure of phage that compromises the ability of biological control. On the other hand, high resistance to temperature increases in the microencapsulated bacteriophages may be due to the protection conferred by the encapsulating material.

UV-irradiation can inactivate phage infectivity and impedes its potential application as biocontrol agents [47]. In our study, microencapsulation provided a protective effect to phage infectivity that was maintained during the exposure time. This UV light-tolerance and temperature resistance observed in microencapsulated bacteriophages could allow phages to remain stable during UV and heat treatments that are usual methods to extend shelf life of some food products [48–50]. In contrast, the free phage cocktail was sensitive to UV exposure as previous studies had demonstrated that UV exposed-non encapsulated bacteriophages could reduce their concentration between 3 and 4 log_10_ or be completely inactivated after 30 min [30,46].

In general, phages are sensitive to acidic media, however, under these conditions the risk of food contamination by pathogenic bacteria is also reduced [17]. In our study, both formulations showed an adverse effect at pH 2, but between pH 3 and 7, microencapsulated phages were more resistant. Coffey and colleagues [51] observed that phages e11/2 and e4/1c against *E*. *coli* reduced their viability to undetectable levels at pH 2, while phage survival was not significantly different at pH values between 3 and 10. Furthermore, Fister and colleagues [44] demonstrated that phage P100 numbers were reduced below detection limit at pH 2, and after 24 h at pH 4–10 viability was no significantly reduced.

For regulation reasons, in case of phage ingestion, it is important to evaluate phage viability through the gastrointestinal tract. Both formulations were sensitive to SGF after 5 min exposure, and stable in bile salt. The reduction in phage survival may be mainly due to denaturation of phage coat proteins caused by the extreme acidity of gastric fluid and it was also clearly observed that for microencapsulated phages, the encapsulating matrix did not provide sufficient protection. Tolerance to biliary secretions has already been shown for lytic free and microencapsulated phages of enteric bacteria which, after 3 h incubation in 1% and 2% bile solutions, reduced their concentration around 1 log_10_ [31]. There were no negative effects on the viability of microencapsulated bacteriophages when exposed to simulated intestinal fluid. *In vivo* assays demonstrated that after oral phage administration in mice, microencapsulated and free phages could be recovered from faeces with minimal impact on intestinal *E*. *coli* concentration. These results are consistent with studies that have demonstrated that phages survive to the gastrointestinal tract and could be recovered from animal faeces in a dose-dependent manner [33,42,52]. Notably, even when microencapsulated and free phages scarcely survive to pH 2 *in vitro*, they were capable to survive their passage through the mouse stomach. It has been previously stated that the mouse stomach pH might reach up to 4.0 and the gastric emptying after a meal or in fasting conditions could vary between T_1/2_ 2 to 16 min [53,54]. All these variables in gastric motility and pH could have allowed the phage survival in our *in vivo* model.

Protein characterization is a necessary step when considering the use of protein-based products for human consumption due to the risk of inducing allergies. Few studies have been conducted to determine the allergenic potential of bacteriophages used as biological control in food, however, none of them has found proteins with potential to cause cross-reactivity or allergy, because it is rare to find true cross-reactivity if the proteins share less than 50% identity over its full-length [55–58]. However, Codex Alimentarius recommends a conservative approach for the analysis, in order to find identities of 35% over 80 amino acids (FAO/WHO criterion), sequence similarities with known allergens, and stability of potential IgE bindings [59]. In our single approach analysis by AllergenOnline, we did not detect phage proteins with cross-reactivity with less than 50% identity over their entire length [19,20], notwithstanding seven proteins did not meet the FAO/WHO criterion. Further, a multicriteria analysis predicting allergenicity using *in silico* tools that explore different approaches were employed for these proteins. Only few bioinformatics tool parameters predicted protein sequences as probable allergens, in general almost all predicted phage protein sequences as non-allergens. Interestingly, six of the seven proteins analysed are probably not structural proteins of the viral particle, thus their probability to act as an allergen is negligible. The last one, the tail fibre adhesion protein, did not show hits in additional bioinformatic analyses, although, a serological test to identify significant overlap of the proteins analysed with some allergenic proteins should be addressed [60,61].

In summary, we demonstrated that microencapsulation of bacteriophages improved survival under the effect of hostile factors as pH, temperature and UV light giving greater stability during storage and application and showed the potential of phages products and warrant future studies.

## Supporting information

S1 Table Log *E*. *coli* concentration on tomato surface. (XLSX)

S2 Table Host range of phage cocktail. (XLSX)

S3 Table Survival percentages of microencapsulated and free phages at -80°C and -20°C storage temperatures. (XLSX)

S4 Table Survival percentages of microencapsulated and free phages at 25°C, 50°C and 75°C storage temperatures. (XLSX)

S5 Table Survival percentages of microencapsulated and free phages exposed to UV light. (XLSX)

S6 Table Survival percentages of microencapsulated and free phages exposed to different pH. (XLSX)

S7 Table Survival percentages of microencapsulated and free phages exposed to SGF. (XLSX)

S8 Table Survival percentages of microencapsulated and free phages exposed to bile salts. (XLSX)

S9 Table Phage faecal shedding after the administration of free or microencapsulated phage cocktails at different doses. (XLSX)

S10 Table Percentage reduction in *E*. *coli* shedding in free or microencapsulated phage cocktail-treated mice. (XLSX)

S11 Table Phage titer calculation. (XLSX)
